# Acute respiratory muscle unloading improves time-to-exhaustion during moderate- and heavy-intensity cycling in obese adolescent males

**DOI:** 10.1038/s41598-020-74240-4

**Published:** 2020-10-12

**Authors:** Hailu Kinfu Alemayehu, Desy Salvadego, Gabriella Tringali, Roberta De Micheli, Mara Caccavale, Alessandro Sartorio, Bruno Grassi

**Affiliations:** 1 Department of Medicine, University of Udine, Piazzale M. Kolbe 4, 33100 Udine, Italy; 2 Department of Physiology, St. Paul’s Hospital Millennium Medical College, Addis Ababa, Ethiopia; 3 Experimental Laboratory for Auxo-Endocrinological Research, Istituto Auxologico Italiano, IRCCS, Milan, Piancavallo (VB) Italy; 4 Division of Metabolic Diseases and Auxology, Istituto Auxologico Italiano, IRCCS, Milan, Piancavallo (VB) Italy

**Keywords:** Metabolism, Physiology, Medical research

## Abstract

Obesity significantly impairs breathing during exercise. The aim was to determine, in male obese adolescents (OB), the effects of acute respiratory muscle unloading, obtained by switching the inspired gas from ambient air (AIR) to a normoxic helium + oxygen gas mixture (HeO_2_) (AIR → HeO_2_) during moderate [below gas exchange threshold (GET)] and heavy [above GET] constant work rate cycling. Ten OB [age 16.0 ± 2.0 years (mean ± SD); body mass index (BMI) 38.9 ± 6.1 kg/m^2^] and ten normal-weight age-matched controls (CTRL) inspired AIR for the entire exercise task, or underwent AIR → HeO_2_ when they were approaching volitional exhaustion. In OB time to exhaustion (TTE) significantly increased in AIR → HeO_2_ vs. AIR during moderate [1524 ± 480 s vs. 1308 ± 408 (*P* = 0.024)] and during heavy [570 ± 306 s vs. 408 ± 150 (*P* = 0.0154)] exercise. During moderate exercise all CTRL completed the 40-min task. During heavy exercise no significant differences were observed in CTRL for TTE (582 ± 348 s [AIR → HeO_2_] vs. 588 ± 252 [AIR]). In OB, but not in CTRL, acute unloading of respiratory muscles increased TTE during both moderate- and heavy-exercise. In OB, but not in CTRL, respiratory factors limit exercise tolerance during both moderate and heavy exercise.

## Introduction

Obesity significantly impairs several aspects of breathing^1–3^. The impairments include an increased demand for ventilation, an increased work of breathing and respiratory muscle inefficiency^1^. In obese subjects resting and exercise tidal breathing occur at low operational lung volumes, thereby increasing prevalence and severity of expiratory flow limitation and the resistive load imposed on the respiratory system. The reduced chest wall compliance, attributable to the excess fat mass on the respiratory wall, and the increased work to be performed against abdominal fat and viscera further increase the work of breathing. Obese patients are characterized by a higher O_2_ cost of breathing^2,4–7^ which contributes, at least in part, to the higher O_2_ cost of exercise (higher pulmonary O_2_ uptake [${\dot{\text{V}}}$ O_2_] for the same work rate)^8–10^, which, in turns, reduces exercise tolerance^4^.

The substitution of helium (He) for nitrogen in normoxic HeO_2_ (21% O_2_–79% He) obtains an inspired gas mixture of lower density and higher viscosity compared to normal air^5^. HeO_2_ breathing during exercise reduces flow turbulence, and for the same pulmonary ventilation it unloads respiratory muscles and reduces the O_2_ cost of breathing and the O_2_ cost of exercise^11^. By reducing flow turbulence, moreover, HeO_2_ breathing reduces also expiratory flow limitation during exercise.

In a previous study by our group^5^ acute respiratory muscle unloading via normoxic HeO_2_ breathing reduced the O_2_ cost of exercise and perceived exertion in a group of obese adolescents (OB) during both moderate- (~ 70% of the gas exchange threshold (GET) and heavy- (~ 120% of GET) intensity short-duration (12 min) exercise on a cycle ergometer. In that study, however, participants did not perform exercise until volitional exhaustion and, as such, the impact of respiratory muscle unloading on exercise tolerance was not directly examined. Moreover, a control group of normal-weight subjects was not involved.

In the present study we evaluated, in OB and in normal-weight controls (CTRL), the acute effects of respiratory muscle unloading, obtained by switching the inspired gas from ambient air (AIR) to normoxic HeO_2_ (AIR → HeO_2_) as patients were approaching exhaustion during long-duration (40 min or up to volitional exhaustion) constant work rate moderate- and heavy-intensity cycling. Time to exhaustion and other variables related to exercise tolerance were determined. We hypothesized an increased time to exhaustion following AIR → HeO_2_ in OB, but not in CTRL, providing further evidence of a respiratory limitation to exercise tolerance in obese adolescents, also during moderate-intensity exercise, in other words in an exercise domain corresponding to activities of daily living. The findings would confirm the need for implementing therapeutic approaches specifically aimed at improving respiratory muscle function in the treatment of obesity.

## Methods

### Subjects

We studied ten male OB [age: 16.0 ± 2.0 years (mean ± standard deviation, SD)], Tanner stage 4–5 (*i.e.* late puberty), who were admitted as in-patients (Division of Auxology, Italian Institute for Auxology, Piancavallo, Italy) for a multidisciplinary body weight reduction program, and ten age- and sex- matched controls (CTRL) [age: 17.0 ± 0.9 years]. Inclusion criteria were: (1) BMI > 97th centile [OB] and < 50th centile [CTRL] for age and sex, using the Italian growth charts12; (2) no involvement in structured physical activity programs (regular activity more than 120 min/week) during the 8 months preceding the study; (3) absence of overt uncompensated diabetes; (4) absence of signs or symptoms referable to any major cardiovascular, respiratory or orthopedic disease contraindicating or significantly interfering with the tests. The standard deviation score (SDS) of BMI was calculated by applying the LMS method [based upon the skewness (L), the median (M), and the coefficient of variation (S) of the measurements as a function of age to Italian reference values for children and adolescents]^12^. Fat-free mass (FFM) was assessed by bioelectric impedance analysis^13^. Whole body resistance to an applied current (50 kHz, 0.8 mA) was measured with a tetrapolar device (Human IM, Dietosystem, Italy). Fat mass (FM) was calculated as the difference between total body mass (BM) and FFM.

Participants agreed to be enrolled in the study. Participants’ parents provided informed signed consent statements, after being fully advised about the purposes and testing procedures of the investigation, which were approved by the ethics committee of the Italian Institute for Auxology, Piancavallo, Italy, where the experiments were carried out. All procedures were performed in agreement with the recommendations set forth in the Helsinki Declaration^14^.

Before the exercise protocols, the OB and CTRL subjects performed pulmonary function testing while breathing room air (Quark CPET, COSMED Srl, Italy). Tests were performed according to the guidelines of the American Thoracic Society^15^. Forced vital capacity (FVC), forced expiratory volume in the 1st second (FEV_1_), FEV_1_/FCV and peak expiratory flow (PEF) were determined. Predicted values were determined based on Hankinson et al.^16^.

### Exercise protocol

Each study participant was seen three times on separate days. All tests were conducted under medical supervision, and study participants were continuously monitored by 12-lead electrocardiography (ECG). A mechanically braked cycle ergometer (Monark Ergomedic 839E) was utilized. Pedaling frequency was digitally displayed to the participants, who were asked to keep a constant cadence throughout the tests between 60 and 70 revolutions per minute. Each participant had chosen his preferred cadence during practice trials; the resistance of the pedals was calculated to obtain the desired work rate^10^.

Participants were allowed time to gain familiarity with the investigators and experimental set up and were familiarized with the exercise protocols (particularly the AIR → HeO_2_ switching) by means of several preliminary practice tests. During the first day the participants performed an incremental exercise test breathing room air. After 3 min of resting measurement (subjects sitting on the cycle ergometer) the incremental exercise began with a 2-min warm-up at 20 W; the work rate was then increased by 20 W every 2 min until the subjects reached volitional exhaustion, defined as the inability to maintain the imposed work rate at the required frequency despite strong verbal encouragement. For all variables values determined at exhaustion were considered “peak” values^10^.

During two subsequent days participants performed two bouts of constant work rate exercises, in AIR or following AIR → HeO_2_ [the switch occurring when the patients were reaching heart rate (HR) values close to those corresponding to volitional exhaustion (see below). By utilizing a 3-way non-rebreathing valve (Hans Rudolph Inc., USA) the subjects inspired from a 180 L Douglas bag continuously primed with air (21% O_2_, 79% N_2_) or with He-O_2_ (21% O_2_, 79% N_2_). Care was taken to ensure that layering in the gas cylinders due to density differences between He and O_2_ did not occur. The inspired gas mixture was humidified. The subjects were blinded with respect to the inspired gas composition. The sequence of conditions (AIR and AIR → HeO_2_) was randomized.

The AIR → HeO_2_ switching occurred when HR reached ~ 85% (for moderate-intensity exercise or ~ 95% (for heavy-intensity exercise) of peak HR. Identification of the percentage of peak HR at which to perform the AIR → HeO_2_ switching was empirical. During preliminary trials it was indeed observed that volitional exhaustion occurred in OB, during moderate exercise, at ~ 85% of peak HR, whereas during heavy exercise the percentage of HR peak at volitional exhaustion was higher (~ 95%). No explanation can be given for this difference. The different durations of the exercise were presumably involved: with longer exercise volitional exhaustion was associated with lower HR values. Work rates were selected to be in the moderate (~ 80% of GET [see below] and in the heavy (~ 50% of the difference between GET and peak ${\dot{\text{V}}}$ O_2_) intensity domains. Moderate-intensity exercise was carried out for 40 min or to volitional exhaustion, whereas heavy intensity exercise was carried out to volitional exhaustion. Moderate intensity exercise was always performed before heavy intensity exercise. About 2 h separated the two exercise intensities; the recovery period was enough for the main investigated variables to reach baseline values.

### Measurements

Pulmonary ventilation (${\dot{\text{V}}}_{{\text{E}}}$), tidal volume (V_T_), respiratory frequency (f_R_), O_2_ uptake (${\dot{\text{V}}}$ O_2_) and CO_2_ output (${\dot{\text{V}}}$ CO_2_) were determined on a breath-by-breath basis by means of a metabolic unit (Quark CPET, COSMED Srl, Italy) when subjects breathed AIR. The greater viscosity of He impacted both the gas analyzers and the pulmonary ventilation measurements by the metabolic unit. In order to take into account these effects complex calibration procedures would be necessary, which could not be performed during the necessarily rapid AIR → HeO_2_ switching. Thus, ${\dot{\text{V}}}_{{\text{E}}}$ and gas exchange measurements during the AIR → HeO_2_ trial were inaccurate, and they were not taken into consideration. Calibration of O_2_ and CO_2_ analyzers was performed before each experiment in AIR by utilizing gas mixtures of known composition (Air Liquide, Milan, Italy). Expiratory flow measurements were performed by a bidirectional turbine, which was calibrated by a 3 L syringe at different flow rates. HR was determined by ECG. Ratings of perceived exertion (RPE) for respiratory discomfort (RPE_R_) and limb effort (RPE_L_) were obtained at rest and every minute during exercise by using the Borg’s modified CR10 scale^17^. GET was determined by the V-slope method proposed by Beaver et al.^18^; ventilatory equivalents (${\dot{\text{V}}}_{{\text{E}}}$/${\dot{\text{V}}}$ O_2_, ${\dot{\text{V}}}_{{\text{E}}}$/${\dot{\text{V}}}$ CO_2_) were utilized as ancillary evidence^18^. The gas exchange ratio (R) was calculated as ${\dot{\text{V}}}$ CO_2_/${\dot{\text{V}}}$ O_2_.

### Statistical analysis

Results were expressed as mean ± standard deviation (SD). Statistical significance of differences between groups was checked by two-tailed Student’s *t*-test for unpaired data. Statistical significance of differences between AIR → HeO_2_ and AIR was checked by two-tailed Student’s *t*-test for paired data. The level of significance was set at *P* < 0.05. Statistical analyses were carried out by utilizing a commercially available software package (Prism 5.0, GraphPad).

## Results

### Physical characteristics of patients and spirometry data

The main anthropometric and spirometry data are reported in Table 1. BM, BMI, BMI SDS, FFM, FM and FM as a percentage of BM were significantly higher in OB vs. CTRL. OB did not show signs of airway obstruction, although FEV_1_/FVC was significantly lower (by 5%) in OB vs. CTRL.

**Table 1:** Anthropometric characteristics and main spirometry data.

	OB	CTRL
BM (kg)	117.5 ± 21.3	68.4 ± 10.0**
Height (m)	1.70 ± 0.12	1.77 ± 0.13
BMI (kg/m^2^)	38.9 ± 6.1	21.4 ± 2.7**
BMI-SDS	3.3 ± 0.3	− 0.2 ± 0.9***
FFM (kg)	71.7 ± 10.5	53.5 ± 6.2**
FM (kg)	45.8 ± 11.3	14.9 ± 4.6**
FM (% of BM)	38.7 ± 2.9	21.5 ± 3.6**
FVC, L	5.0 ± 0.7	5.6 ± 1.1
FVC, % predicted	107.1 ± 10.7	108.4 ± 19.0
FEV_1_, L	4.3 ± 0.8	4.9 ± 0.9
FEV_1_, % predicted	105.5 ± 16.2	112.4 ± 21.6
FEV_1_/FVC, %	84.7 ± 5.8	89.7 ± 4.6*
FEF_25–75%_	4.6 ± 1.3	5.4 ± 1.2
FEF_25–75_, % predicted	102.7 ± 28.3	94.3 ± 19.5
PEF, L/s	8.0 ± 1.3	8.3 ± 2.4
PEF, % predicted	95.8 ± 6.5	100.1 ± 23.4

*OB* obese adolescents, *CTRL* control subjects, *BM* body mass, *BMI-SDS* SD score of body mass index (BMI), *FFM* fat free mass, *FM* Fat mass, *TBW* total body water, *FVC* forced vital capacity, *FEV*_*1*_ forced expiratory volume in 1 s, *FEF*_*25%–75%*_ forced expiratory flow between 25 and 75% of FVC, *PEF* peak expiratory flow. Values are expressed as mean ± standard deviation.

***P < 0.001; **P < 0.01; *P < 0.05.

### Incremental exercise

Peak values of the main investigated variables obtained during the incremental exercise test are reported in Table 2. Peak values of work rate, HR and ${\dot{\text{V}}}_{{\text{E}}}$ were significantly lower in OB vs. CTRL. Peak ${\dot{\text{V}}}$ O_2_ values were ~ 20% lower in OB vs. CTRL when expressed in L min^−1^. After normalizing peak ${\dot{\text{V}}}$ O_2_ values per unit of BM, or per unit of LBM, values in OB were ~ 100% and ~ 40% lower, respectively, than those in CTRL. On the other hand GET values, when reported as a percentage of peak work rate (72.1 ± 6.0% [OB] vs. 71.9 ± 9.9% [CTRL]) or of peak ${\dot{\text{V}}}$ O_2_ (73.0 ± 6.0% [OB] vs. 76.3 ± 7.3% [CTRL]) were not significantly different in OB vs. CTRL.

**Table 2:** Peak values of the main variables determined during the incremental exercise test.

	OB	CTRL
Exercise duration, s	972 ± 182	1224 ± 241*
Work rate, watt	164 ± 32	206 ± 40*
HR, beats/min	170.6 ± 9.0	189.1 ± 4.5***
RPE_R_ (0–10)	6.3 ± 3.1	8.3 ± 2.1
RPE_L_ (0–10)	8.0 ± 2.1	9.2 ± 1.0
SpO_2_, %	97.4 ± 1.6	96.3 ± 2.1
${\dot{\text{V}}}$ O_2_, L/min	2.48 ± 0.48	3.03 ± 0.63*
${\dot{\text{V}}}$ O_2_, mL/min/kg	21.2 ± 4.0	44.6 ± 9.9***
${\dot{\text{V}}}$ CO_2_, L/min	2.62 ± 0.58	3.29 ± 0.66*
R	1.04 ± 0.05	1.09 ± 0.04
${\dot{\text{V}}}_{{\text{E}}}$, L/min	86.4 ± 25.4	115.5 ± 24.2*
PETO_2_, mmHg	94.6 ± 3.9	116.4 ± 5.1***
PETCO_2_, mmHg	34.2 ± 3.4	33.4 ± 5.7
f_R_, breaths/min	37.9 ± 5.7	49.2 ± 10.6**
V_T_, L	2.3 ± 0.5	2.4 ± 0.4

*OB* obese adolescents, *CTRL* control subjects, *HR* heart rate, *RPE*_*L*_ rate of perceived exertion for leg effort, *RPE*_*R*_ rate of perceived exertion for respiratory discomfort, *SpO*_*2*_ arterial blood O_2_ saturation by pulse oximetry; $\dot{V}$ *O*_*2*_ **O_2_ uptake, $\dot{V}$ *CO*_*2*_ CO_2_ output, *R* respiratory gas-exchange ratio, $\dot{V}_{E}$ pulmonary ventilation, PETCO_2_, CO_2_ end-tidal pressure; PETO_2_, O_2_ end tidal pressure, *f*_*R*_ respiratory frequency, *V*_*T*_ tidal volume; Values are expressed as mean ± standard deviation.

***P < 0.001; **P < 0.01; *P < 0.05.

### Constant work rate exercise

Results of the main variables obtained at the end of moderate- and heavy-intensity constant work rate exercise are shown in Table 3.

**Table 3:** Main variables determined at the end of moderate- and heavy-intensity constant work rate exercise in AIR and in AIR + HeO_2_.

	Moderate intensity			Heavy intensity			
	OB AIR	CTRL AIR	OB AIR → HeO_2_	OB AIR	CTRL AIR	OB AIR → HeO_2_	CTRL AIR → HeO_2_
Exercise duration, s	1308 ± 408***	2400 ± 0	1524 ± 480 §	408 ± 150	588 ± 252	570 ± 306 §	582 ± 348
Work rate, watt	97.0 ± 23.3	116.8 ± 23.9	97.0 ± 23.3	142.5 ± 30.5*	176.0 ± 33.4	142.5 ± 30.5	176.0 ± 33.4
Work rate, % peak	59 ± 5	57 ± 8	59 ± 5	87 ± 3	86 ± 5	87 ± 3	86 ± 5
HR, beats/min	146.9 ± 13.4*	163.2 ± 15.0	148.2 ± 15.5	160.1 ± 11.4***	183.9 ± 5.5	165.4 ± 12.2	182.8 ± 5.6
HR, % peak	86 ± 5	86 ± 8	87 ± 6	94 ± 3	97 ± 4	97 ± 3	97 ± 3
RPE_R_	4.1 ± 3.3	4.8 ± 3.5	3.3 ± 3.4	4.5 ± 3.1**	8.1 ± 1.8	3.8 ± 3.5	6.6 ± 2.6
RPE_L_	8.1 ± 2.3	7.3 ± 2.5	6.4 ± 2.9 §	8.8 ± 1.8	9.7 ± 0.5	8.0 ± 2.6	9.8 ± 0.4
${\dot{\text{V}}}$ O_2_, L/min	1.83 ± 0.40*	2.30 ± 0.30		2.30 ± 0.50**	3.00 ± 0.50		
${\dot{\text{V}}}$ O_2_, % peak	74 ± 7	77 ± 10		93 ± 6*	100 ± 6		
${\dot{\text{V}}}$ O_2_, mL/kg/min	15.5 ± 2.5***	33.9 ± 4.8		19.6 ± 3.3***	44.5 ± 8.3		
${\dot{\text{V}}}$ CO_2_, L/min	1.68 ± 0.40*	2.02 ± 0.26		2.31 ± 0.53**	2.99 ± 0.49		
R	0.91 ± 0.05	0.88 ± 0.03		0.99 ± 0.05	1.00 ± 0.05		
${\dot{\text{V}}}_{{\text{E}}}$, L/min	55.3 ± 17.2	63.4 ± 7.0		78.2 ± 23.0**	111.3 ± 18.7		
f_R_, breaths/min	32.6 ± 5.0	34.5 ± 5.8		38.5 ± 6.0*	48.8 ± 10.4		
V_T_, L	1.7 ± 0.4	1.9 ± 0.3		2.0 ± 0.4	2.3 ± 0.3		
PETO_2_, mmHg	91.5 ± 4.0***	106.0 ± 5.1		94.9 ± 3.9***	114.7 ± 3.7		
PETCO_2_, mmHg	34.1 ± 3.2	37.6 ± 5.3		33.2 ± 2.9	31.4 ± 4.2		

*OB* obese adolescents, *CTRL* control subjects, *HR* heart rate, *RPE*_*L*_ rate of perceived exertion for leg effort, *RPE*_*R*_ rate of perceived exertion for respiratory discomfort, $\dot{V}$ *O*_*2*_ O_2_ uptake, $\dot{V}$ *CO*_*2*_ CO_2_ output, *R* respiratory gas-exchange ratio, $\dot{V}_{E}$ pulmonary ventilation, *f*_*R*_ respiratory frequency, *V*_*T*_ tidal volume, *PETCO*_*2*_ CO_2_ end-tidal pressure, *PETO*_*2*_ O_2_ end tidal pressure. Values are expressed as mean ± standard deviation.

***P < 0.001; **P < 0.01; *P < 0.05 (vs. CTRL AIR). § = *P* < 0.05 vs. OB AIR.

During moderate intensity exercise all CTRL completed the 40 min exercise task, whereas OB reached exhaustion significantly earlier (21 min 48 s ± 6 min 48 s). As a consequence of the completion of the task by all subjects, during moderate intensity exercise no AIR → HeO_2_ switching was performed by CTRL. During moderate intensity exercise in OB time to exhaustion increased by ~ 15% (*P* < 0.05) in AIR → HeO_2_ (25 min 24 s ± 8 min) vs. AIR. Despite the longer exercise duration in AIR → HeO_2_, end-exercise HR values were not significantly different between the two conditions.

During heavy intensity exercise in AIR OB reached exhaustion earlier (6 min 48 s ± 2 min 30 s) than CTRL (9 min 48 s ± 4 min 2 s), although the difference did not reach statistical significance (*P* = 0.065). During heavy intensity exercise time to exhaustion in OB increased by ~ 40% (*P* < 0.05) in AIR → HeO_2_ (9 min 30 s ± 5 min 6 s) vs. AIR; also in this case HR was not significantly different between the two conditions. During heavy intensity exercise in CTRL no significant differences were observed for the time to exhaustion (9 min 42 s ± 5 min 42 s in AIR → HeO_2_ vs. in AIR); no significant difference was observed also for HR.

In OB the AIR → HeO_2_ switching occurred on average after 22 min 30 s during moderate exercise, and after 7 min 42 s during heavy exercise; in CTRL the switching occurred after 8 min 30 s during heavy exercise.

**Figure 1:**
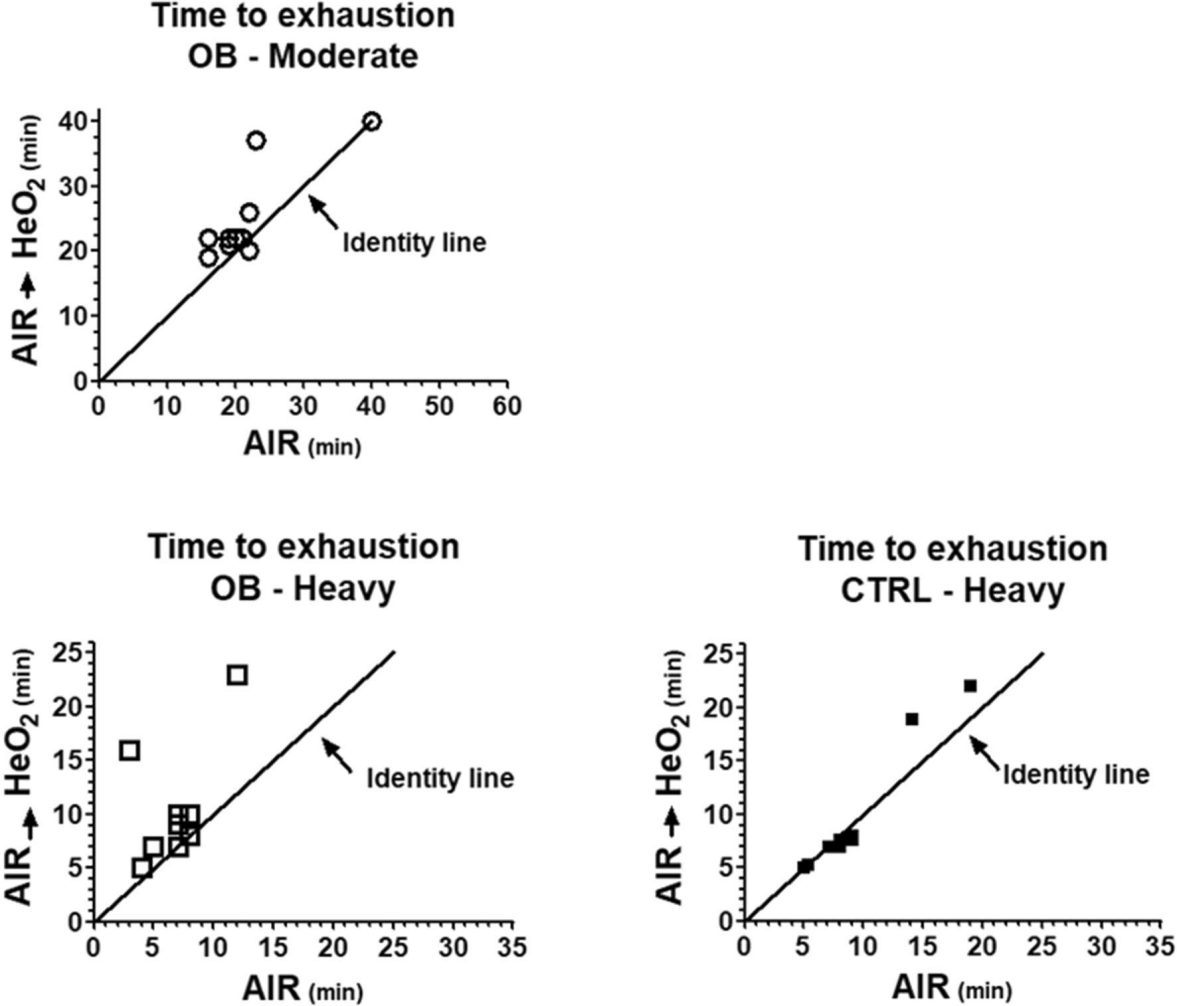
Individual values of time to exhaustion are presented as a scatter plot, for the two group of subjects (obese patients, OB; healthy controls, CTRL), for moderate- and heavy-intensity exercise in the two experimental conditions (AIR and AIR → HeO_2_). Time to exhaustion values in AIR are shown on the *x* axis, whereas time to exhaustion values in AIR → HeO_2_ are shown on the *y* axis. The identity lines are also shown. As discussed in the text, similar figure could not be made for moderate exercise in CTRL, since all subjects completed the 40-min exercise task in AIR, and no AIR → HeO_2_ switching was performed. See text for further details.

Individual values of time to exhaustion are presented as a scatter plot in Fig. 1. Time to exhaustion values in AIR are shown on the *x* axis, whereas time to exhaustion values in AIR → HeO_2_ are shown on the *y* axis. The identity line is also presented. In OB, both during moderate and during heavy exercise all individual data points, with the exception of two, appear above the identity line. This means that in most OB patients time to exhaustion was longer in AIR → HeO_2_ vs. in AIR. On the other hand for all CTRL subjects, with the exception of two, individual data points appear on the identity line, or slightly below it. Thus, in most CTRL subjects time to exhaustion was lower or unchanged in AIR → HeO_2_ vs. in AIR. As mentioned above, a similar figure could not be made for moderate exercise in CTRL, since all subjects completed the 40-min exercise task in AIR and no AIR → HeO_2_ switching was performed.

**Figure 2:**
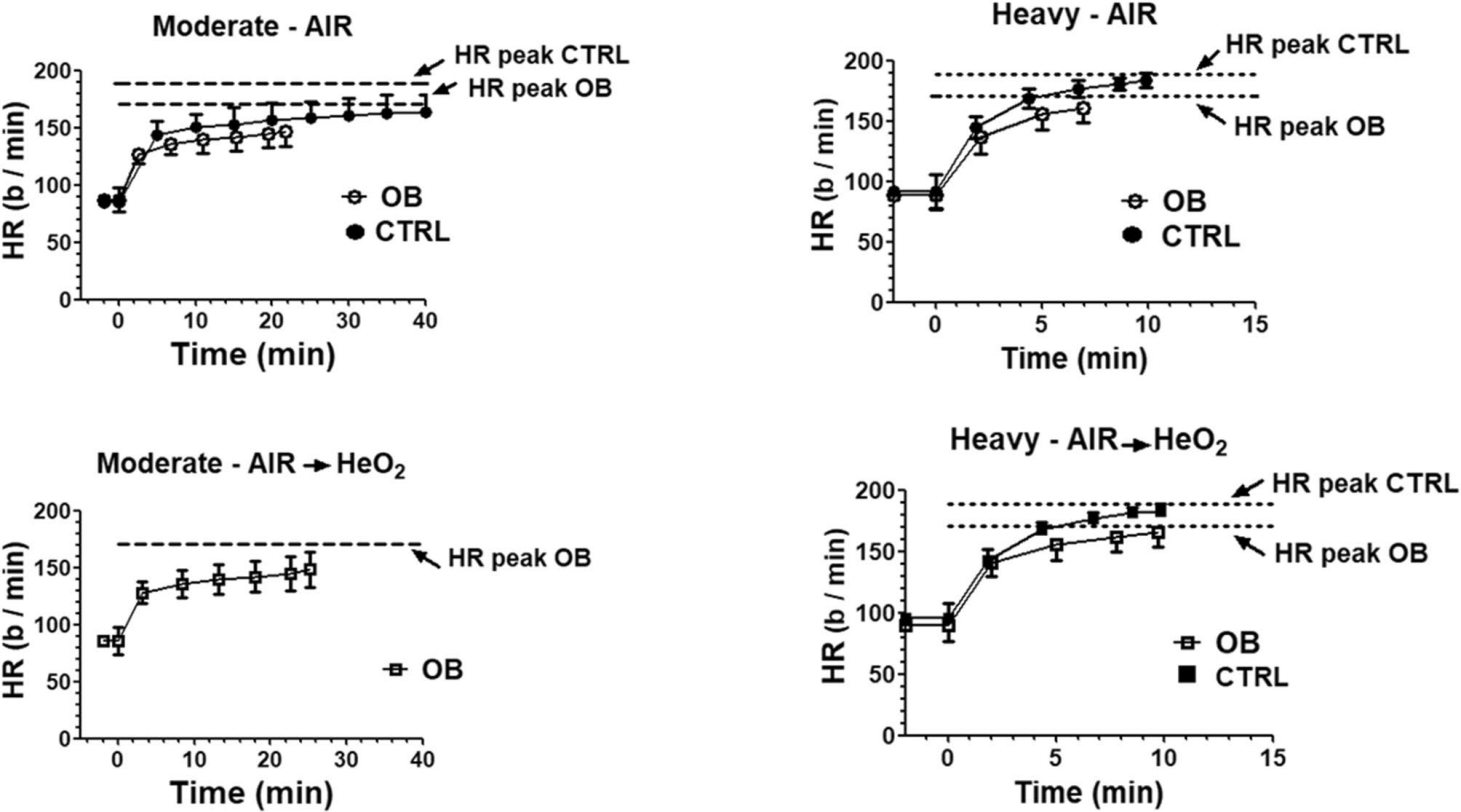
Mean ± standard deviation heart rate (HR) values calculated every minute during moderate-intensity and heavy-intensity exercise, up to volitional exhaustion (or to 40 min for moderate-intensity exercise in CTRL) in the two group of subjects (obese patients, OB; healthy controls, CTRL), in the two experimental conditions (AIR and AIR → HeO_2_). The horizontal dashed lines indicate the peak HR values determined during the incremental exercise. See text for further details.

Figure 2 shows the HR vs. time patterns for OB and CTRL during moderate- and heavy- intensity exercise. To obtain this figure, in order to take into account the progressive “selection”, as a function of exercise duration, of subjects with higher times to exhaustion, individual values were grouped for discrete time intervals; the time intervals were chosen in order to have, in each interval, each subject represented by one data point. When the subject had more than one "original" data point in the interval, mean individual values were calculated, both for the x and the y variables. The resulting HR patterns were similar in both conditions (AIR and AIR → HeO_2_) and in both groups of subjects. A clear evidence of a slow component of the HR kinetics was indeed observed, during both moderate- and heavy- intensity exercise and in both experimental conditions, and no evidence of a steady-state of the variable was present. Peak HR values obtained at exhaustion during the incremental test are also shown in the Figure. During heavy intensity exercise both OB and CTRL reached exhaustion in the presence of HR values not significantly different from those determined at exhaustion (peak HR) during the incremental test. On the other hand, during moderate intensity exercise OB patients reached exhaustion in the presence of HR values well below those corresponding to peak HR.

## Discussion

In the present study we demonstrated that in OB the acute unloading of respiratory muscles, obtained by switching the inspired gas from AIR to normoxic HeO_2_ (AIR → HeO_2_) when the subjects were approaching exhaustion, significantly increased time to exhaustion during both moderate and heavy intensity exercises. Time to exhaustion, on the other hand, did not increase following AIR → HeO_2_ in CTRL. Thus, in OB, respiratory limitations contribute to the impaired exercise tolerance observed also during moderate intensity exercise, corresponding to daily-living activities.

In a previous study by our group^5^ acute respiratory muscle unloading obtained by normoxic He-O_2_ breathing reduced the O_2_ cost of exercise and perceived exertion in a group of obese adolescents, during both moderate- and heavy-intensity short-duration (12 min) constant work rate exercise on a cycle ergometer. Although a reduced O_2_ cost of exercise and a reduced perceived exertion should be associated with an increased exercise tolerance, in that study^8^ this variable was not directly evaluated by the determination of time to exhaustion. The main aim of the present work was to fill this gap, and to directly demonstrate the increased exercise tolerance following respiratory muscle unloading.

All CTRL subjects completed the 40-min trial of moderate intensity exercise. Thus, the findings of the present study are consistent with the well-established concept that relatively long moderate intensity exercise in healthy humans does not cause significant fatigue^4,8^. This, however, did not occur in OB, as these patients reached volitional exhaustion well before the 40-min time limit, also after the AIR → HeO_2_ switch. Moderate-intensity exercise and GET, therefore, may have quite different meanings in OB with respect to normal subjects, and OB may reach fatigue and exhaustion relatively early also during moderate-intensity exercise. Only in part this limitation in OB would be related to respiratory factors associated to the increased work of breathing: after eliminating or reducing these factors by the AIR → HeO_2_ switch, indeed, time to exhaustion increased in OB by a few minutes, but it remained substantially lower than in CTRL.

The fatigue and the exhaustion observed in OB during moderate intensity exercise do not seem to be related to the “slow component” of variables such as HR (see Fig. 2), as hypothesized in a previous study by Zuccarelli et al.^19^. A clear HR slow component during moderate intensity exercise was indeed present both in OB and in CTRL, but fatigue and premature exhaustion occurred only in OB. Moreover, OB reached exhaustion during moderate intensity exercise in the presence of HR values below those (peak HR) reached at exhaustion during the incremental test. On the other hand, it cannot be excluded that the HR slow component may only matter, in terms of fatigue development, when other factors related to the OB “condition” are present.

The issue of fatigue and exhaustion, occurring in OB also during moderate intensity exercise, seems of significant relevance for its impact on exercise tolerance during activities associated with daily living, as well as on exercise prescription, and deserves further investigations, aimed at identifying the mechanism(s) responsible for the premature fatigue, and the variables allowing its evaluation.

In CTRL the AIR → HeO_2_ switch did not affect exercise performance during heavy intensity cycling (see Fig. 1), thereby supporting the concept that the respiratory factors do not limit exercise tolerance at submaximal intensities in healthy and normally active subjects^20^.

The mechanisms responsible for the improvement of exercise tolerance achieved by the AIR → HeO_2_ switch in OB could involve complex interactions between respiratory and locomotor muscle, in relation to O_2_ delivery, as well as among respiratory, locomotor muscle and central nervous system in relation to voluntary activation^21–23^. In short, through a “metaboreflex” respiratory muscle fatigue would decrease skeletal muscle activation by the central nervous system, leading to exhaustion. Respiratory muscle unloading by HeO_2_ breathing or by respiratory muscle endurance training^6,7^, however, would not only interrupt or attenuate the metaboreflex described above, but would also positively affect “central” hemodynamics”^7,24^ and enhance O_2_ delivery to locomotor muscle. Within a general *scenario* of a limited cardiac output in relation to competing needs by different organs and systems, respiratory muscle unloading could allow to divert blood flow from respiratory to locomotor muscle, thereby reducing the O_2_ cost of locomotor muscle and further increasing exercise tolerance^22,25–27^. Enhanced blood flow and O_2_ delivery to locomotor muscle would indeed go in the direction of delaying or reducing the development of peripheral muscle fatigue, maintaining metabolic stability in these muscles^4^ and preventing the recruitment of additional less efficient muscle fibers^28^. Maintenance of metabolic stability and prevention of a decreased muscle efficiency would be inherently associated with less muscle fatigue^4^.

In normal subjects interventions aimed at modifying the work of breathing during near-maximal whole-body exercise significantly affect both respiratory and quadriceps muscle blood flow^25^: when the work of breathing was decreased (by a proportional assist ventilator), respiratory muscle blood flow decreased and quadriceps blood flow increased; this phenomenon was associated with an increased exercise performance^26^. On the other hand, when the work of breathing was increased (by resistors), respiratory muscle blood flow increased and quadriceps blood flow decreased^25^. These observations support the concept of a “competition” between respiratory and quadriceps muscle for blood flow during intense exercise, occurring also in normal-weight healthy subjects. In obese patients, a population characterized by higher work of breathing and by an increased metabolic demand as a consequence of the greater body mass, this phenomenon could be present also during moderate-intensity exercise.

Respiratory muscle unloading via normoxic HeO_2_ extended exercise duration in patients with heart failure^29^ and increased lower limb muscle oxygen delivery during heavy-intensity exercise in chronic obstructive pulmonary disease (COPD) patients^30,31^. Studies carried out on COPD patients^24,31^ observed that a reduced expiratory muscle activity obtained by HeO_2_ breathing may also determine improvements in cardiac output and central hemodynamics, the net result being an increased peripheral muscle O_2_ delivery.

The present study has several limitations. All CTRL carried out moderate intensity exercise to the time-limit (40 min) set in the protocol, and therefore no AIR → HeO_2_ switching could be performed by these subjects during moderate intensity exercise. The subjects would have presumably continued this exercise for hours before reaching exhaustion^4^; this further strengthens the difference with respect to OB, who reached exhaustion in ~ 22 min.

As mentioned above, since we could not perform the calibration procedures of the metabolic cart necessary to take into account the greater viscosity of HeO_2_ compared to that of normal air, pulmonary ventilation and gas exchange variables could not be determined during the AIR → HeO_2_ trial. No analysis of respiratory mechanics was carried out, and no direct measurement or estimation of the work of breathing were performed.

The redistribution of blood flow from respiratory to locomotor muscle could be only hypothesized, on the basis of literature data^25,26^, and was not directly demonstrated. Insights into this redistribution could derive from non-invasive measurements of fractional O_2_ extraction (O_2_ uptake/O_2_ delivery) by near-infrared spectroscopy (NIRS)^32^ in locomotor and respiratory muscles. NIRS measurements in skeletal muscle, however, are virtually impossible in obese patients, as a consequence of the increased thickness of the subcutaneous fat layer^32^.

The present study was carried out only on male subjects and patients. Sex-differences have been described for the inspiratory muscle metaboreflex^33^. More specifically, activation of the metaboreflex by fatigue of inspiratory muscle would determine less pronounced cardiovascular consequences in females vs. males^33^. No inferences can be made about female obese adolescents.

To conclude, in the present study, following an acute unloading of respiratory muscles, obtained by the AIR → HeO_2_ switching as the patients were approaching exhaustion, male OB could increase time to exhaustion during both moderate- and heavy-intensity constant work rate exercise. This occurred although the time to exhaustion values in OB, also after the AIR → HeO_2_ switch, were still substantially lower than those observed in CTRL. The improvement in exercise tolerance following the AIR → HeO_2_ switch was not observed in CTRL. Thus, in male OB respiratory muscle represents a “weak spot” among the physiological adaptations to exercise; we demonstrated that this occurs also during moderate intensity exercise, corresponding to daily-living activities. These findings confirm the need for implementing therapeutic approaches specifically aimed to improve the performance of respiratory muscle in the treatment of male obese adolescents; an answer could derive from respiratory muscle endurance training, as suggested by two previous studies by our group^6,7^. By contrasting the vicious circle of: obesity → early fatigue → reduced exercise tolerance → reduced physical activity → obesity, the intervention could represent a useful adjunct in the control of obesity in male adolescent patients.

## Data Availability

The datasets generated and/or analyzed during the current study are available from the corresponding author on reasonable request.
